# Laryngopharyngeal reflux in chronic obstructive pulmonary disease - a multi-centre study

**DOI:** 10.1186/s12931-020-01473-2

**Published:** 2020-08-21

**Authors:** Julia Sanchez, Desiree M. Schumann, Meropi Karakioulaki, Eleni Papakonstantinou, Frank Rassouli, Matthias Frasnelli, Martin Brutsche, Michael Tamm, Daiana Stolz

**Affiliations:** 1 Clinic of Respiratory Medicine and Pulmonary Cell Research, University Hospital Basel, Petersgraben 4, CH-4031 Basel, Switzerland; 2 Department of Pneumology, Kantonsspital St. Gallen, Rorschacherstrasse 95, St. Gallen, CH-9001 Switzerland; 3 Department of Pneumology, Kantonsspital Graubünden, Loestrasse 170, Chur, CH-7000, Chur Switzerland

**Keywords:** Laryngopharyngeal reflux, Chronic obstructive pulmonary disease, Proton pump inhibitor therapy, RYAN score, Gastroesophageal reflux

## Abstract

Reflux of gastric content has been associated with recurrent exacerbations of chronic obstructive pulmonary disease (COPD). We aimed to assess the prevalence of laryngopharyngeal reflux (LPR) in COPD and if LPR is a contributing factor to clinically relevant outcomes in COPD. We evaluated a total of 193 COPD patients (GOLD I-IV) with a 24-h laryngo-pharyngeal pΗ-monitor. LPR was observed in 65.8% of COPD patients and it was not significantly associated with clinically relevant outcomes of COPD. Treatment with PPI significantly decreased the upright RYAN score (*p* = 0.047) without improving lung function. Furthermore, the presence or severity of LPR cannot be diagnosed based solely on symptoms and questionnaires.

## Background

Chronic obstructive pulmonary disease (COPD) is a chronic respiratory disease and one of the leading causes of mortality worldwide [1]. The clinical severity of COPD is determined by comorbidities, one of which is the gastroesophageal reflux disease (GERD) [2–5]. GERD is a common cause of chronic cough [6] and a potential risk factor for exacerbations of COPD [7–10]. Frequent exacerbators have a high prevalence of GERD, however approximately 58% of these patients lack typical GERD symptoms [11, 12].

Laryngopharyngeal reflux (LPR) represents an extra-esophageal manifestation of GERD. The reflux of gastric contents is fundamental in both LPR and GERD, but the mechanism and the symptoms of the disorders are distinct [13–15]. LPR occurs when gastric contents pass the upper esophageal sphincter and usually occurs during daytime in the upright position, while GERD occurs when gastric contents pass the lower esophageal sphincter and takes place more often in the supine position at night-time or during sleep [16]. LPR may be a contributing factor in patients with symptomatic COPD however, there are only a few studies analyzing the impact of LPR in patients with COPD [13, 17, 18]. In a large longitudinal study of COPD patients, self-reported GERD or use of PPIs was associated with a 20–60% increased risk of moderate-severe exacerbations and hospitalized exacerbations during 3 years of follow up [19]. Yet, this study was based on a subjective, self-reported history of a physician’s diagnosis of GERD and studies based on objective evaluations by laryngeal-pharyngeal pH monitoring in a large COPD cohort are still missing. Here, we investigated the prevalence of LPR and explored its association with clinically relevant outcomes of COPD.

## Methods

We included 193 patients with mild to severe COPD (GOLD I-IV). All patients completed the GERD questionnaire (GerdQ) and the Reflux symptom index (RSI), in order to assess reflux symptoms and the Leicester cough questionnaire, in order to assess life quality disturbance due to cough. Additionally, patients were evaluated for the prevalence of LPR and its association with lung function. The presence of LPR was assessed by trained and certified study nurses. Participants were fitted with a pharyngeal probe (Restech pH probe, Respiratory Technology Corp.) for 24 h. The pH was measured twice per second and was transmitted wirelessly to a data recorder. The thresholds for detecting LPR were for the upright position: RYAN score > 9.41 and for the supine position: RYAN score > 6.81 [20].

A subgroup of 107 patients belonged to a prospective, multicenter study [PREVENT, ISRCTN 45572998 [21, 22]] and was longitudinally assessed for 2 years (median follow-up period of 12 months) for an association between LPR and clinically relevant outcomes of COPD. In this pre-defined cohort, 41 patients had 67 mild acute exacerbations of COPD (AECOPD), defined as an acute worsening of respiratory symptoms leading to a change in medication and 37 patients had 62 severe AECOPD, requiring hospitalization. Among these 107 patients, 34 patients agreed to undergo a second evaluation of LPR after 1 month on PPI treatment (Supplementary Figure 1).

## Results

The descriptive characteristics of the patients are presented in Table 1A. Risk categories were defined for 106 of the 107 patients from the pre-defined cohort as follows: 8 in GOLD A, 63 in GOLD B, 4 in GOLD C and 31 in GOLD D.

**Table 1:** [A]: Descriptive characteristics of the patients included in the study; [B]: Linear regression model for the effect of various parameters in the upright RYAN score in the pre-defined cohort with 2-year follow-up

**[A]**				
**Descriptive characteristics**	**All patients** *n* = 193		**Pre-defined cohort with 2-year follow-up** *n* = 107	
**Age (years)**, mean (SD)	66.2 (8.8)		67.5 (8.4)	
**BMI (kg/m**^**2**^**)**, mean (SD)	27.1 (6.7)		27.8 (6.6)	
**Medical history of GERD,** n (%)	33 (17)		16 (15)	
**Treated with PPI,** n (%)	69 (37)		33 (30.8)	
**Gender,** male (%)	119 (62)		75 (70)	
**Smoking status,** n (%)				
Current	62 (34)		38 (36)	
Past	121 (66)		69 (64)	
**GOLD Stage,** n (%)				
I	25 (14)		6 (6)	
II	82 (45)		58 (58)	
III	55 (30)		32 (32)	
IV	21 (11)		4 (4)	
**COPD Medication**				
LABA	55 (28)		19 (18)	
LABA+ICS	151 (78)		102 (96)	
LAMA	129 (67)		81 (76)	
SABA	26 (13)		2 (2)	
SAMA	39 (20)		35 (33)	
**Lung Function (post-BD),** mean (SD)				
FEV_1_%predicted	56.9 (21.8)		57.4 (16.4)	
RV % predicted	144.3 (49.4)		136.6 (46.7)	
TLC %predicted	110.5 (20.5)		107.9 (20.5)	
DLCO% predicted	61.0 (22.8)		57.7 (18.5)	
FEV_1_/FVC	46.9 (14.1)		46.9 (13.1)	
**Questionnaire scores,** mean (SD)				
GerdQ	2.1 (3.3)		2.1 (3.2)	
Leicester cough	96.5 (38.0)		104.6 (27.1)	
RSI	10.0 (9.2)		10.1 (8.4)	
**[B]**				
**Parameter**	**Pre-defined cohort with 2-year follow-up** (*n* = 107)			
	**Beta**	**95% CI: Lower**	**95% CI: Upper**	***p*-value**
**Age**	0.239	0.006	0.050	**0.013**
**6MWT**	−0.073	−0.002	0.001	0.474
**BODE Index**	0.004	−0.099	0.103	0.969
**Lung Function**				
FEV_1_% predicted	−0.010	−0.013	0.012	0.923
TLC % predicted	−0.182	−0.019	0.001	0.091
RV % predicted	−0.142	−0.007	0.001	0.177
**Νumber of exacerbations during the study**	0.015	−0.175	0.205	0.877
**Number of severe exacerbations during the study**	−0.011	−0.305	0.272	0.911
**Questionnaires**				
GerdQ	−0.257	−0.139	− 0.022	**0.008**
Leicester cough	0.012	−0.007	0.008	0.899
RSI	−1.013	−0.034	0.011	0.314

*95% CI* 95% confidence interval, *BMI* body mass index, *GERD* gastroesophageal reflux disease*, PPI* proton pump inhibitors, *GOLD* Global Initiative for Chronic Obstructive Lung Disease, *LABA* long acting beta 2 agonist, *LABA + ICS* long-acting beta 2 agonist plus glucocorticosteroids, *LAMA* long-acting muscarinic antagonist, *SABA* short-acting beta 2 agonist, *SAMA* short-acting muscarinic antagonist, *post-BD* post-bronchodilator, *FEV*_*1*_ forced expiratory volume in 1 s, *RV* residual volume, *TLC* total lung capacity, *DLCO* diffusing capacity of the lung for carbon monoxide, *GerdQ* gastroesophageal reflux disease questionnaire, *RSI* Reflux symptom index, *BODE Index* Body mass, airflow obstruction, dyspnea and exercise capacity index, *6MWT* 6-min walking test

**Fig. 1:**
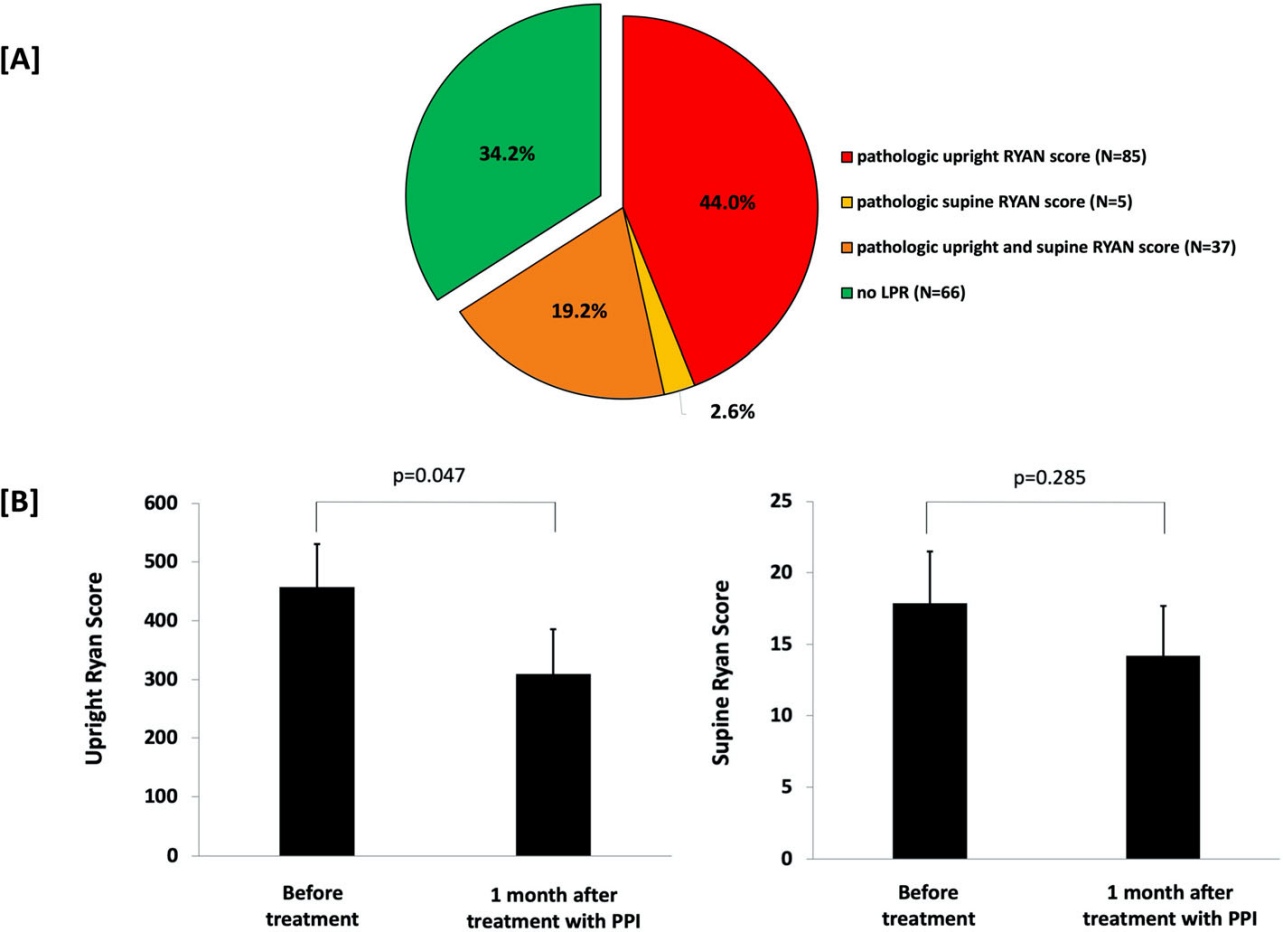
[**a**] Prevalence of LPR in COPD patients. [**b**] RYAN score in upright and supine position before treatment and after 1-month of proton-pump inhibitor (**PPI**) treatment in 34 COPD patients. Bars represent the mean ± standard error of mean

The median (IQR) upright RYAN score was 37.01 (2.12–186.89) and the median (IQR) supine RYAN score was 2.17 (2.17–5.50). Totally, 65.8% (*n* = 127) of the patients had LPR, as detected by either a pathologic upright RYAN score (*n* = 85, 44.0%), or a pathologic supine RYAN score (*n* = 5, 2.6%), or both upright and supine pathologic RYAN scores (*n* = 37, 19.2%) (Fig. 1a).

**Fig. 2:**
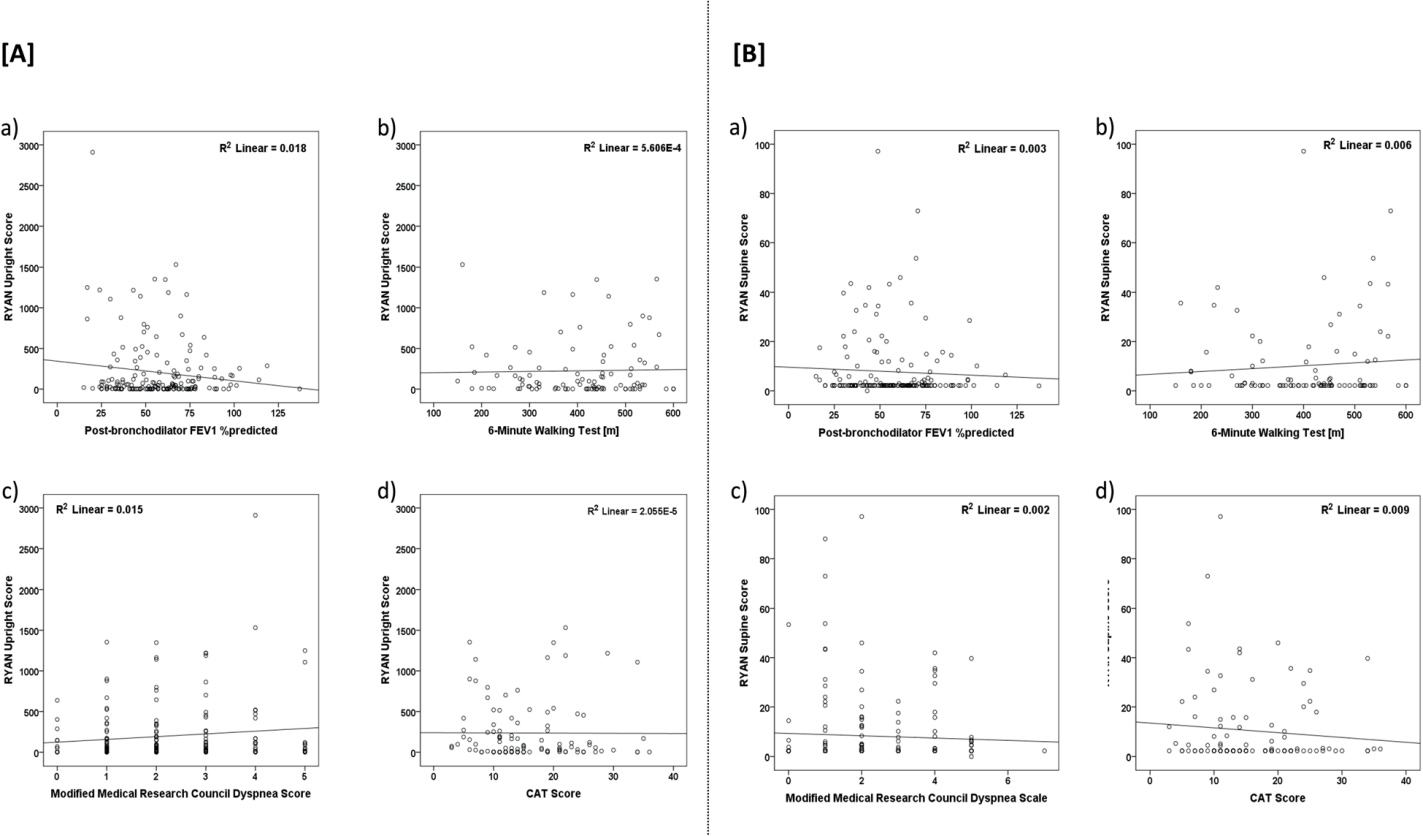
[**A**] Correlation between RYAN upright score and a) post-bronchilator FEV1%predicted; b) 6-min walking test; c) Modified Medical Research Council dyspnea score and d) COPD Assessment Test (CAT) score [**B**] Correlation between RYAN supine score and a) post-bronchilator FEV1%predicted; b) 6-min walking test; c) Modified Medical Research Council dyspnea score and d) CAT score

There was no association between upright or supine RYAN score and lung function measurements (FEV_1_% predicted, *p* = 0.076 and *p* = 0.488, respectively; RV % predicted, *p* = 0.282 and *p* = 0.800, respectively; TLC % predicted, *p* = 0.054 and *p* = 0.559, respectively; Fig. 2a and b).

In the pre-defined cohort with 2-years follow-up, linear regression analysis revealed that there was no association between the upright RYAN score and COPD severity, as revealed by the 6-min walking test, the BODE index, and lung function (Table 1B). Furthermore, there was no association between the upright RYAN and COPD outcome as revealed by the number of exacerbations during the study (Table 1B). Similar results were also obtained for the supine RYAN score (Fig. 2b). However, there was a significant positive association between the upright RYAN score and age (Beta = 0.239, *p* = 0.013), and a negative association with GerdQ (Beta = − 0.257, *p* = 0.008) (Table 1B).

There were no significant differences between patients with positive LPR and patients with negative LPR in GerdQ score (1.9 ± 3.3 vs 2.1 ± 3.3, *p* = 0.177), Leicester cough score (93.9 ± 40.0 vs 103.3 ± 32.8, *p* = 0.227) and RSI (9.9 ± 9.0 vs 9.4 ± 9.6, *p* = 0.905).

Using Cox regression and adjusting the model for age and PPI therapy, we found no effect of upright RYAN score neither on time to exacerbation (Exp(B) 1.325, *p* = 0.369) nor on time to severe exacerbation (Exp(B) 1.195, *p* = 0.722).

Within the pre-defined cohort with 2-year follow-up, 34 COPD patients were reevaluated for LPR, after 1 month on PPI treatment. There was a significant decrease in the upright RYAN score after treatment (*p* = 0.047) but not in the supine RYAN score (*p* = 0.285) (Fig. 2b). Comparing lung function before and after 1 month on PPI treatment, we found no significant difference in lung function parameters, or in any of the questionnaire scores.

## Discussion

To our knowledge, this is the largest study assessing LPR prevalence in a well-characterized COPD cohort. We assessed LPR by monitoring laryngopharyngeal pH [19] and we could demonstrate that the prevalence of LPR is high in COPD patients (65.8%). This is in agreement with the study of Hamdan et al. [13], where the RSI questionnaire was utilized to determine the presence of LPR in 27 COPD patients and 67% of them scored positive for LPR. In our study, there was no association between LPR and clinically relevant COPD outcomes within a 2-year follow-up period, contrary to the findings of Jung et al. [17], who found an association between the RSI score, the reflux finding score and severe exacerbations in 118 COPD patients. These discrepancies could be attributable to the fact that Jung et al. [17] refrained from objectively analyzing LPR prevalence and their diagnosis was based solely on symptoms. Additionally, as stated by Jung et al. [17], the respiratory symptoms of COPD, such as excess throat mucus, cough, throat clearing, and dysphonia, coincide with the measurements of the RSI questionnaire. Therefore, during an exacerbation of COPD, these symptoms would increase automatically, resulting in an increase in the RSI score, independently of LPR. Jung et al. [17] found no association between RSI, RFS, and COPD severity or any other lung function parameter, except between RFS and residual volume / total lung capacity (%).We found no association between the questionnaire results (GerdQ-Questionnaire, RSI to assess reflux symptoms and the Leicester cough questionnaire to assess lifetime quality disturbance due to cough) and RYAN score results which confirms findings in other studies [23, 24].

Currently, the main treatment for LPR is PPI. We found that in patients receiving PPI for 30 days, there was a significant decrease in the upright RYAN score but no improvement in lung function nor in symptoms, as assessed by the various questionnaires used. Further long-term investigations are required to clarify this finding though these results have been seen with GERD treatment [25] and LPR treatment for 2-months in COPD patients [18]. The findings are also in-line with the recent ERS guidelines on chronic cough, suggesting that PPI therapy is not beneficial for patients with reflux without dyspeptic symptoms [26].

Among the limitations of our study is the short follow-up period after PPI therapy. However, it was possible to determine a significant decrease in the upright RYAN score after 1 month on PPI. Within the strengths of our study is its multicentric design and the large well-characterised COPD cohort included.

## Conclusions

LPR is not significantly associated with clinically relevant outcomes of COPD. Treatment with PPI significantly decreased the upright RYAN score without improving lung function. Our results further indicate that the presence or severity of LPR cannot be diagnosed based solely on symptoms and questionnaires.

## Supplementary information

**Additional file 1.**

## Data Availability

The datasets used and analysed during the current study are available from the corresponding author on reasonable request.

## Abbreviations

AECOPD: Acute exacerbations of COPD
BODE index: Body mass, airflow obstruction, dyspnea and exercise capacity index
CI: Confidence interval
CAT: COPD Assessment Test
COPD: Chronic obstructive pulmonary disease
DLCO: Diffusing capacity of the lung for carbon monoxide
FEV_1_: Forced expiratory volume in 1 s
FVC: Forced vital capacity
GerdQ: Gastroesophageal reflux disease questionnaire
GERD: Gastroesophageal reflux disease
GOLD: Global initiative for chronic obstructive lung disease
LPR: Laryngopharyngeal reflux
PPI: Proton pump Inhibitors
RSI: Reflux symptom index
RV: Residual volume
TLC/RV: Residual volume to total lung capacity ratio
TLC: Total lung capacity
