# Supplementary figures and images for: Laryngopharyngeal reflux in chronic obstructive pulmonary disease - a multi-centre study
Source: Respir Res. 2020 Aug 21;21:220. doi: 10.1186/s12931-020-01473-2 (PMC7441701; doi:10.1186/s12931-020-01473-2)

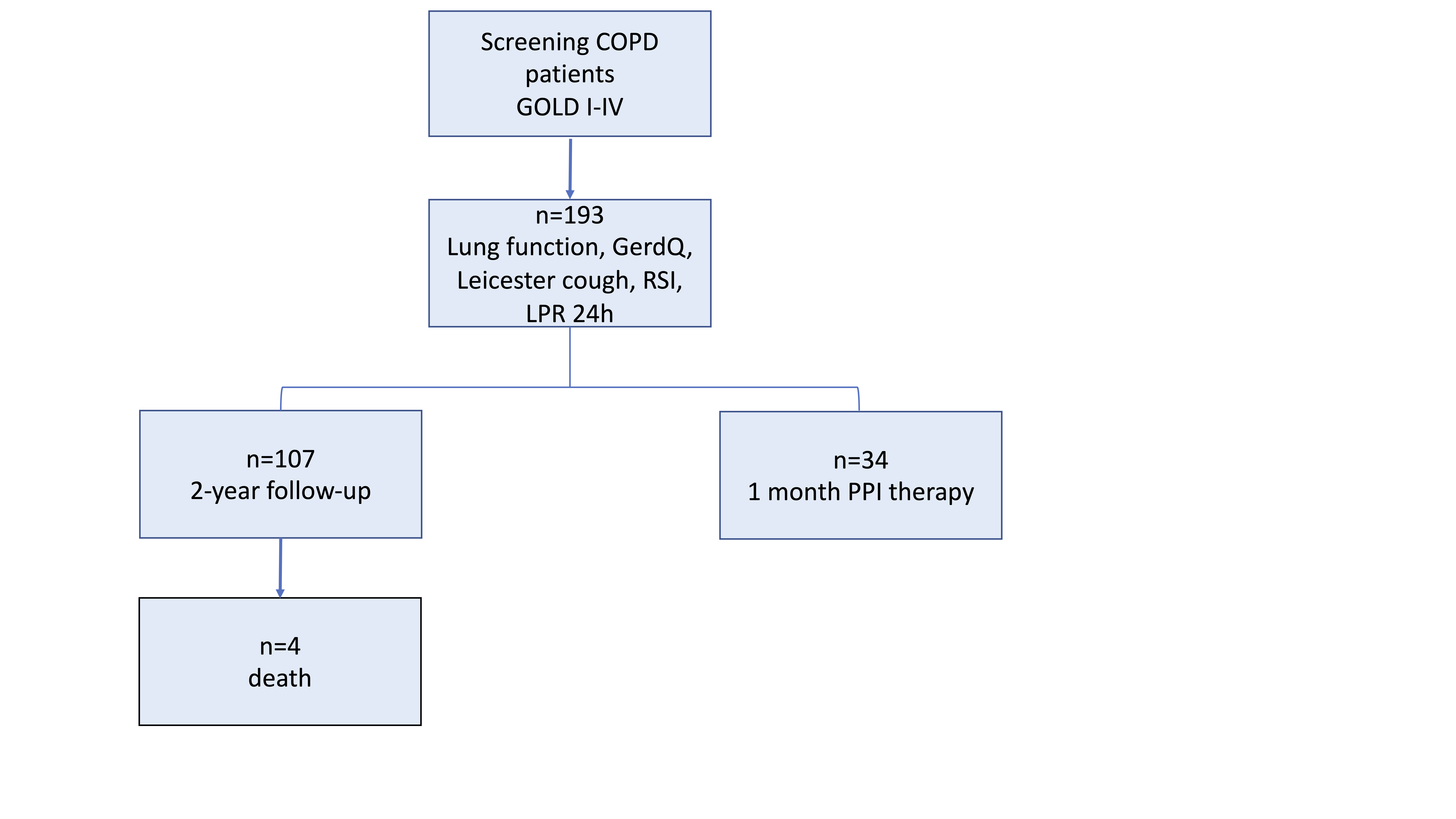

Supplement: Supplementary file 1 — Additional file 1. [file 12931_2020_1473_MOESM1_ESM.jpg]
